# Electrophoretic Deposition of Carbon Nanotubes on 3-Amino-Propyl-Triethoxysilane (APTES) Surface Functionalized Silicon Substrates

**DOI:** 10.3390/nano3020272

**Published:** 2013-05-13

**Authors:** Anirban Sarkar, Theda Daniels-Race

**Affiliations:** Division of Electrical and Computer Engineering, School of Electrical Engineering and Computer Science, Louisiana State University, 3101 P. F. Taylor Hall, Baton Rouge, LA 70803, USA; E-Mail: asarka4@lsu.edu

**Keywords:** carbon nanotubes, electrophoretic deposition (EPD), APTES (3-aminopropyl-triethoxysilane), surface functionalization, acid reflux

## Abstract

Fabrication of uniform thin coatings of multi-walled carbon nanotubes (MWCNTs) by electrophoretic deposition (EPD) on semiconductor (silicon) substrates with 3-aminopropyl-triethoxysilane (APTES) surface functionalization has been studied extensively in this report. The gradual deposition and eventual film formation of the carbon nanotubes (CNTs) is greatly assisted by the Coulombic force of attraction existing between the positively charged –NH_2_ surface groups of APTES and the acid treated, negatively charged nanotubes migrating towards the deposition surfaces. The remarkable deposition characteristics of the CNT coatings by EPD in comparison to the dip coating method and the influence of isopropyl (IPA)-based CNT suspension in the fabricated film quality has also been revealed in this study. The effect of varying APTES concentration (5%–100%) on the Raman spectroscopy and thickness of the deposited CNT film has been discussed in details, as well. The deposition approach has eliminated the need of metal deposition in the electrophoretic deposition approach and, therefore, establishes a cost-effective, fast and entirely room temperature-based fabrication strategy of CNT thin films for a wide range of next generation electronic applications.

## 1. Introduction

The invention of helical microtubules of graphitic carbon by Iijima in 1991 [1] by arc-discharge evaporation has stimulated stupendous research endeavors in the wondrous world of carbon nanotubes (CNTs) all over the world. Investigation of the chemical, mechanical and electrical properties and optimization of different parameters in the growth models of the nanotubes have been the state-of-art research in nano-science and technology over the previous decades [2,3]. In the field of next generation consumer electronics, substantial studies have been pursued into the device integration of carbon nanotubes on various substrates with the existing complementary metal-oxide-semiconductor (CMOS) technology, thereby exploring numerous potential applications, like CNT-based thin film transistors, field emission devices, nano-electromechanical systems, display systems, energy storage, flexible electronics, and so forth [4,5,6,7,8,9,10]. The conventional direct growth models of single walled and multi-walled carbon nanotubes (SWCNTs and MWCNTs) by arc-discharge, laser ablation and chemical vapor deposition (CVD) are limited by the high temperature processing, presence of unwanted metal impurities and soot-like carbonaceous skeins in the final nanotube yield and the need for expensive vacuum systems [11,12,13]. Especially, the high temperature growth techniques offer a serious bottleneck in the integration of carbon nanotubes directly for some applications, like low temperature microelectromechanical systems (MEMS), flexible, plastic electronics and molecular electronics. Therefore, in recent years, a lot of attention has converged in the development of room temperature, low cost, solution-based CNT wet coating techniques [14] to tune CNT integration compatible with the said applications. Numerous innovative deposition methods from different stable CNT suspensions have been, so far, introduced, such as the Langmuir-Blodgett (LB) method, self-assembly of functionalized CNTs on chemically modified substrates, dip coating, drop casting, electrostatic spray, spin coating, inkjet printing, Mayer Rod coating, laser assisted deposition, like MAPLE, and so on [15,16,17,18,19,20,21,22,23,24,25].

Electrophoretic deposition (EPD) [26,27] has been considered as one of the reliable, inexpensive and efficient solution-based CNT coating techniques in this respect. Considered to be a well-established ceramic processing technique only until the early 1990s, the applications of EPD have been expanded to explore a broad range of advanced materials, including biocompatible hydroxyapatite coating, multilayer composites, silica, titanium dioxide and functionally graded novel ceramics in the last 20 years of materials science [28,29]. The mechanism of electrophoretic deposition can be generally conceptualized by two steps. In the first step, known as the electrophoresis, charged particles that are dispersed in a suitable solvent or an aqueous medium migrate towards the desired electrode, due to the application of a DC electric field across the suspension. In the second step, referred to as the deposition step, the particles coagulate and adhere to the electrode surface, thereby forming a coherent deposit eventually. Since there has been no universal consensus in explaining the deposition kinetics of the particles on different substrates by this process, several theories have been adopted to date. Those are: (1) flocculation by particle accumulation especially on porous membranes, as proposed by Hamaker *et al*.; (2) particle charge neutralization process at the deposition electrode or the deposit, as proposed by Grillon *et al*.; (3) electrochemical particle coagulation mechanism, due to the reduction of the repulsive forces between the particles, as proposed by Koelmans; and (4) electrical double layer (EDL) distortion and thinning mechanism, as illustrated by Sarkar and Nicholson [30,31,32,33].

The electrophoretic deposition of a wide array of nanoparticles, including carbon nanotubes, has been, so far, performed mostly on metal or metal coated substrates, such as stainless steel, aluminum, nickel, titanium and glass plates with conductive coatings [34]. The applicability of electrophoretic deposition of carbon nanotubes in microelectronics and microelectromechanical systems (MEMS) can be multiplied profoundly through its deposition accomplishments on different semiconductor substrates, like silicon. In this respect, our research group recently investigated the deposition characteristics of MWCNTs on patterned metal films atop silicon substrates and insulating layers, such as silicon dioxide and silicon nitride [35]. The observed selective deposition and preferential adhesion of CNTs only on the metal film was attributed to the hydrophilic surface reaction between the acid-treated CNTs and the interacting metal surfaces. The effect of hydrophilic interaction was further demonstrated by insufficient coating and extremely poor adhesion of CNTs on bare silicon and insulator surfaces without any surface functionalization. The work was accomplished by the combination of physical vapor deposition process of metal evaporation on the semiconductor substrates followed by the solution based electrophoretic deposition technique. The present study reveals significant progress in the electrophoretic deposition of CNT films on silicon substrates, which were functionalized by 3-aminopropyl-triethoxysilane (APTES) self-assembling monolayer prior to the deposition step. The state-of-art deposition procedures on APTES functionalized substrates, e.g., glass have been predominantly followed in immersion and dip coating, drop casting and layer-by-layer assembling methods [36,37,38]. However, the reliability of these processes in regards to the deposition parameters, packing density and homogeneity of the deposit on the intended surfaces still remains a challenge to be addressed in great details. Additionally, fabrication of multi-layer films with precise control over the thickness and reproducibility of the results add to the bottlenecks of these methods. In this research, the ingenuity of electrophoretic deposition technique in the formation of uniform CNT coating has been suitably extended in conjunction with the self-assembling APTES surface treatment.

The fabrication strategy set forth in our study have eliminated the need of physical vapor deposition of metal films in the deposition model and have, thus, established an entirely solution-based, reproducible and relatively quick route of electrophoretic deposition of CNTs on semiconductor substrates. We have also demonstrated the benefits of using polar solvents, like isopropyl alcohol (IPA), as the EPD suspension in comparison to aqueous medium. The relevant sections of this report reveal extensive details pertaining to the preparation of CNT suspension for the intended EPD process, APTES self-assembly by the hydroxylation and silanization technique and different comparative studies between CNT-IPA and CNT-water suspension in regards to deposition quality and the benefits of EPD over dip coating methods with varying concentration of APTES.

## 2. Results and Discussion

The acid refluxing of the as-purchased carbon nanotubes attaches carboxylic groups (–COOH) on the surface of the tubes, which impart negative surface charges. The resultant electrostatic repulsion between the surface charges prevents inter-tubular agglomeration and ensures appreciable stability of the CNT suspension all throughout the deposition experiments. Additionally, dissolution of unwanted residual metal catalysts in the as-purchased nanotube powder, purification and shortening of the nanotubes are also accomplished by the acid-heat treatment on the nanotubes [39,40].

**Figure 1:**
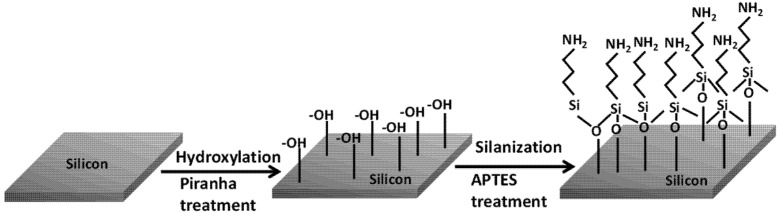
Schematic diagram displaying the process of hydroxylation after piranha treatment and silanization by 3-aminopropyl-triethoxysilane (APTES) treatment on silicon surface prior to the electrophoretic deposition (EPD) step.

The surface functionalization process on the silicon substrates is accomplished by the self-assembly of APTES monolayer, which is triggered by the piranha treatment. The piranha solution introduces abundant surface hydroxyl groups (–OH) on the silicon samples by hydroxylation process. The subsequent silanization step proceeds with the hydrolysis of ethoxy (C_2_H_5_) groups in APTES, leading to the formation of silanols (Si–O–H). The APTES silanols then start to condense with surface silanols, thereby, self-assembling into a monolayer of APTES by a lateral siloxane (Si–O–Si) network, as shown in Figure 1. As can be seen in the siloxane structure, the self-assembled siloxane networks are oriented in such a way that the positively charged amine groups (–NH_2_) are aligned away from the underlying silicon substrate.

**Figure 2:**
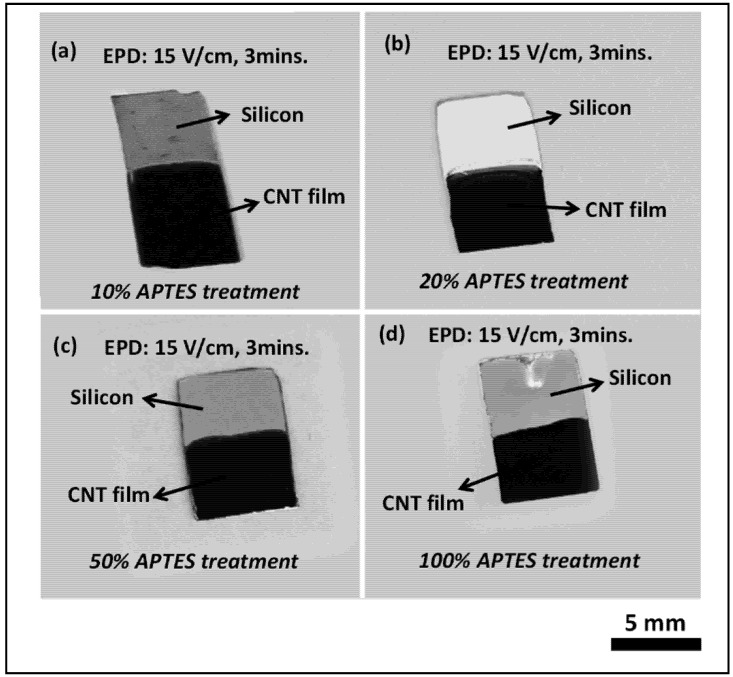
Optical images of carbon nanotube (CNT) film deposited on silicon samples by EPD with different APTES concentration at an applied E-field of 15 V/cm for 3 min (**a**) 10% APTES, (**b**) 20% APTES, (**c**) 50% APTES and (**d**) 100% APTES. All the samples exhibit remarkable film quality without any discontinuity or agglomeration in the morphology.

Figure 2 depicts the electrophoretic deposition results on surface functionalized silicon surfaces with different APTES concentrations (10%, 20%, 50% and 100%) from CNT-IPA suspension. As can be observed from the figure, the electrophoretic deposition of CNT film in conjunction with self-assembled amine terminated monolayer shows remarkable visual homogeneity and packing density in the film quality. Optical microscope observation (100× magnifications) also revealed appreciable surface coverage without any microscopic void or post-drying cracks in the film structure.

The surface functionalized silicon surface (silicon acts as the anode, as it is connected to the positive terminals of the DC power source) imparts sufficient electrophoretic mobility to the nanotubes in the EPD suspension to overcome the inter-tubular force of repulsion and migrate towards the anode surface. The gradual coagulation of the coherent deposit of the nanotubes and eventual film formation is greatly facilitated by the Coulombic force of attraction acting between the positively charged –NH_2_ groups from APTES siloxane structure and the negatively charged nanotubes migrating towards the anode surface. This is the most significant difference with respect to our previous published work, where the thermally evaporated metal layer assisted in adhesion of the nanotubes with the underlying silicon surface. It is assumed that the metal layer acts a glue layer, which binds the carbon nanotubes with the silicon surface. In the present case, the Coulombic interaction between the self-assembled –NH_2_ moieties and –COOH groups on the nanotube surface ensures uniform film formation, thus eliminating the need of metal layer from the deposition model.

Additionally, the piranha and APTES treatment turns the silicon surfaces more hydrophilic. The pristine CNTs are also rendered hydrophilic in the acid refluxing step. The remarkable homogeneity and surface coverage by the CNT deposit in all our EPD experiments can also be attributed to the much improved hydrophilic-hydrophilic interaction between the APTES functionalized depositing surface and the migrating CNTs towards it.

**Figure 3:**
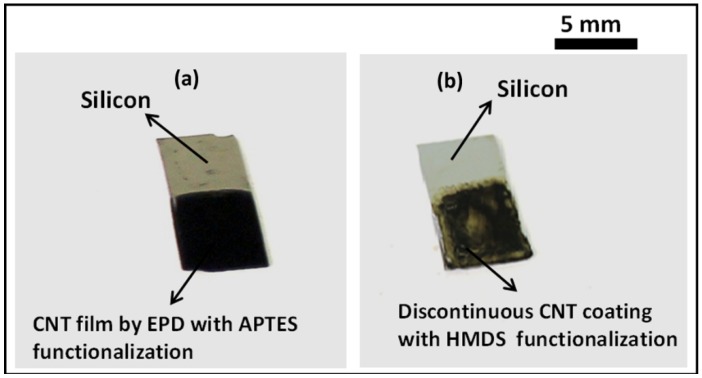
Optical images of CNT films deposited by electrophoretic deposition at an applied E-field of 15 V/cm for 3 min on (**a**) 20% APTES treated silicon sample showing superior coating quality in contrast to (**b**) methyl (–CH_3_) terminated hexamethyldisilazane (HMDS) treated silicon surfaces.

The beneficial effect of hydrophilic APTES self-assembly process in the deposition mechanism is further adjudicated by comparing with methyl (–CH_3_) terminated hexamethyldisilazane (HMDS) functionalization process. The presence of the non-polar methyl group in the hexamethyldisiloxane (–O[Si(CH_3_)_3_]_2_) structure after the HMDS priming treatment renders a surface hydrophobic. The silicon samples were subjected to HMDS coating vapor deposition process at 90 °C for 30 min. Electrophoretic deposition process was then performed on the samples at 15 V/cm for 3 min. Figure 3 shows the difference in coating quality between the APTES treated (left) and HMDS treated silicon (right) samples. In contrast to excellent deposition results on the APTES modified samples, the HMDS coated samples showed discontinuous, inhomogeneous CNT coating with agglomerated deposits. The results, thus, conclusively establishes the beneficial effect of –NH_2_ surface functionalization on the silicon samples in the deposition mechanism when compared with non-polar methyl surface modification, which hinders appreciable adhesion and film formation, due to its inherent hydrophobicity.

**Figure 4:**
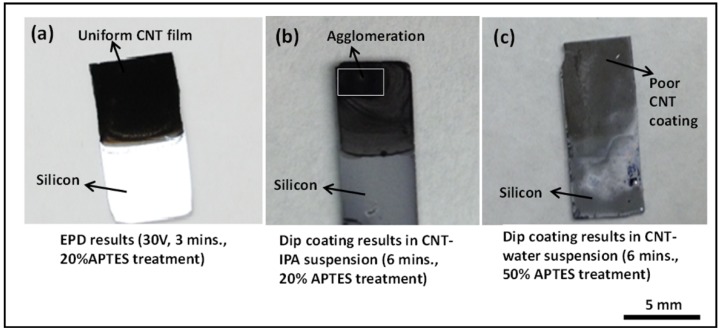
Deposition results comparing electrophoretic deposition (EPD) and dip coating on surface functionalized silicon substrates (**a**) CNT film deposited by EPD at an applied E-field of 15 V/cm for 3 min with 20% APTES treatment, (**b**) dip coated silicon samples in CNT-isopropyl (IPA) solution for 6 min with 20% APTES treatment showing agglomerated deposit and (**c**) dip coated silicon samples results in CNT-water solution for 6 min with 50% APTES treatment showing poor CNT surface coverage.

Having established the benefits of APTES functionalization in the EPD technique, we explored the benefits of APTES assisted electrophoretic deposition in conjunction with APTES assisted dip coating deposition of CNTs. The silicon samples were treated with APTES with varying concentration and dipped in CNT-IPA and CNT-water suspension for 3 min. The electrophoretically deposited CNT films from the same CNT-IPA suspension in all our experiments show superior film quality in comparison to dip coated samples. The dip coated samples in both the CNT-IPA and CNT-water suspension with 20% APTES functionalization for 3 min exhibit poor deposition results with discontinuous and non-uniform CNT coating. The deposition results on CNT-water dip coated samples for 3 min with 20% APTES treatment are worse than the CNT-IPA dip coated samples for the same duration. With a view to improve the deposition characteristics of the dip coated samples, the dip coating duration was increased from 3 to 6 min for both types of the samples, and the APTES treatment of the sample dipped in the CNT-water suspension was increased from 20% to 50%. Figure 4 depicts the experimental results of this comparative study. The results matched with our previous observation in which the CNT-IPA dip coated samples exhibited relatively better deposition characteristics in contrast to CNT-water dip coated samples. Figure 4c shows extremely poor coating characteristics on 50% APTES functionalized CNT-water dip coated samples. The CNT-IPA dipped sample with 20% APTES functionalization still shows inhomogeneous and agglomerated coating features when compared to EPD coated samples (15 V/cm for 3 min). The results presented above clearly represents APTES assisted EPD technique as a better and reliable deposition technique compared to the dip coating method, even with longer incubation duration (6 min of dip coating with 20% APTES functionalization *vs*. 3 min of EPD at 15 V/cm from CNT-IPA suspension with 20% APTES functionalization) and surface treatment with increased APTES concentration (6 min of dip coating with 50% APTES from CNT-water *vs*. 20% APTES assisted EPD from CNT-IPA).

**Figure 5:**
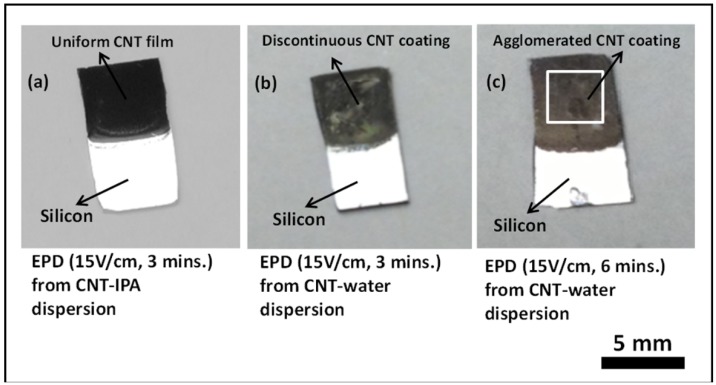
Optical images comparing the electrophoretic deposition results at an applied E-field of 15 V/cm on 20% APTES treated silicon samples from (**a**) CNT-IPA suspension for 3 min, (**b**) CNT-water suspension for 3 min and (**c**) CNT-water suspension for 6 min.

Comparative studies on the CNT films deposited from CNT-IPA and CNT-water suspension by the proposed electrophoresis mechanism were also conducted in this work. As depicted by Figure 5, the deposition results from CNT-IPA suspension for 3 min at 15 V/cm with 20% APTES characterization show superior deposit quality than CNT-water suspension under identical conditions. The samples subjected to CNT-water suspension showed enough agglomeration of the CNTs after 2 h of drying following the deposition step. The results did not improve even though the deposition duration from CNT-water suspension was increased from 3 min to 6 min at 15 V/cm. The reason can be primarily attributed to the generation of bubbles in the solution by the electrolysis of water, due to the strong electric field across the solution. The evolution of hydrogen and oxygen gases by the electrolysis phenomenon disrupts the flow of the CNTs and creates voids and discontinuities in the final deposit at the anode. Similar observation was also reported by Du *et al*. [41] and Boccaccini *et al*. [42] in their attempts to study the morphology of the carbon nanotube films by electrophoretic deposition. Recent literature reports in this direction have revealed the possibility of controlling the rate of electrolysis in aqueous suspension by performing EPD under modulated electric fields, such as pulsed direct current (PDC) and alternating current (AC) [43,44].

The deposition quality also did not improve much when the deposition voltage was reduced to 5 V for longer duration of deposition (30 min), since the resultant electric field (2.5 V/cm) was too low to offer sufficient electrophoretic mobility to the CNTs to migrate and adhere to the anode surface. Additionally, it was also observed that EPD attempts with longer deposition duration from CNT-water suspension sometimes resulted in the total degradation of the solution, leading to formation of agglomerated CNT flakes and subsequent precipitation of the nanotubes in the solution. On the contrary, similar deposition attempts with CNT-IPA solution rarely showed degradation of the solution, although the surface roughness of the final deposit increased, due to prolonged deposition process on the depositing surface.

### 2.1. Microscopic Imaging and Raman Spectroscopy

**Figure 6:**
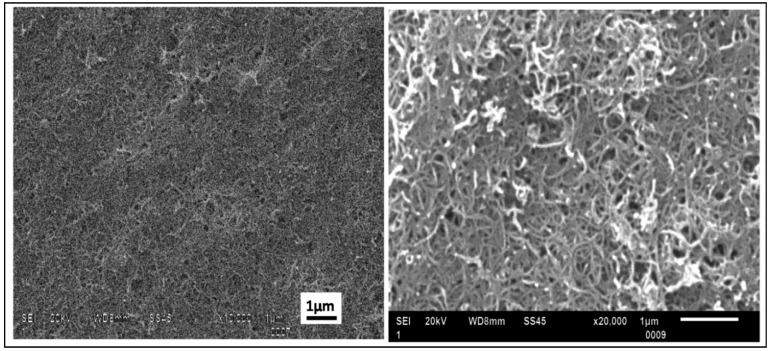
Scanning electron microscopic images of CNT film on 20% APTES treated silicon sample deposited at an applied electric field of 15 V/cm for 3 min showing appreciable surface coverage and packing density without voids (**a**) at ×10 k magnification and (**b**) at ×20 k magnification.

Microstructural imaging of the deposited films was conducted using a JOEL JSM 6610 scanning electron microscopy (SEM) with an acceleration voltage of 20–30 kV. The samples were sputter coated with ~10 nm of platinum before the imaging. Figure 6 shows SEM images of the CNT film deposited on the 20% APTES coated silicon substrates. The images testify to the excellent homogeneity and packing density without any microscopic cracks or discontinuity in the film structure.

**Figure 7:**
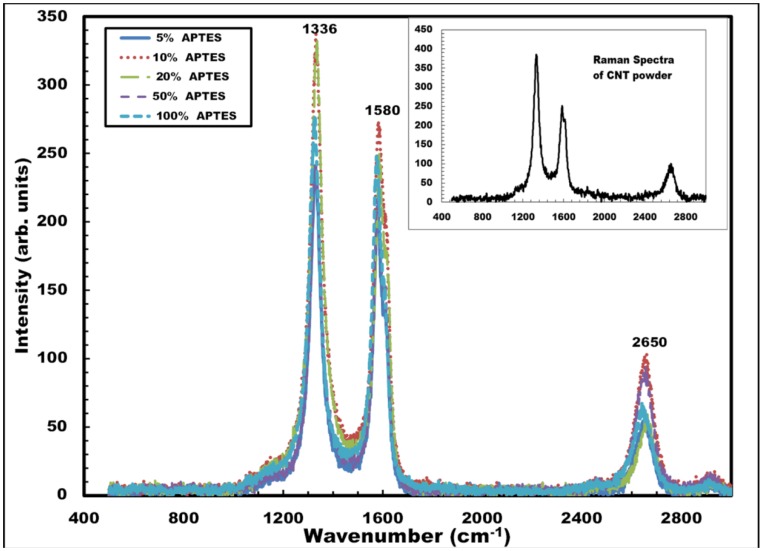
Raman spectra of CNT films deposited on silicon samples with varying APTES concentration (5%, 10%, 20%, 50% and 100%). Inset shows Raman spectrum of raw CNT powder used in the work.

The deposited CNT films were further analyzed by Raman spectroscopy. The spectroscopic measurements were performed using a Jobin Yvon Horiba Labram Raman spectrometer. A HeNe laser with a wavelength of 638.4 nm and an incident power of 17 mW was used for all the spectroscopic analysis. The confocal hole aperture of 200 µm and grating of 1800 lines per mm were selected during the measurement. Extended scans were performed between 500 and 3000 cm^−1^ for best results. The effect of APTES concentration (5%, 10%, 20%, 50% and 100%) in the spectroscopic behavior of the CNT films was extensively studied in the scans. Figure 7 shows the Raman spectra of the carbon nanotube films deposited on the silicon sample with different APTES concentration. The peak for the disorder-induced D-band was seen to occur at ~1333 cm^−1^ and those for the tangential G-bands occurred at ~1579 cm^−1^ and ~2645 cm^−1^ for all the samples. The peaks closely matched with the spectroscopic results of dry carbon nanotube powder used in this work. The absence of radial breathing modes (RBM) was also noted for all the samples when scanning was performed from 0 to 400 cm^−1^ (not shown in the graph). The spectroscopic results, therefore, establish the following: (a) the presence of multi-walled nanotubes in the deposited CNT films, since the absence of the prominent radial breathing modes (RBM) is noted for all the Raman spectra (between 0 and 400 cm^−1^), and (b) the concentration of APTES used in the surface functionalization procedure does not influence the spectroscopic behavior of the deposited CNT films.

### 2.2. Thickness

To explore the effect of varying APTES concentration (5%, 10%, 20%, 50% and 100%) on the quality of the deposited films, quantitative analysis of the film thickness was performed using a KLA-Tencor D-100 Alpha Step surface profiler. For the thickness profiling, surface scanning was performed 10 times for each sample, and the average value was calculated.

**Figure 8:**
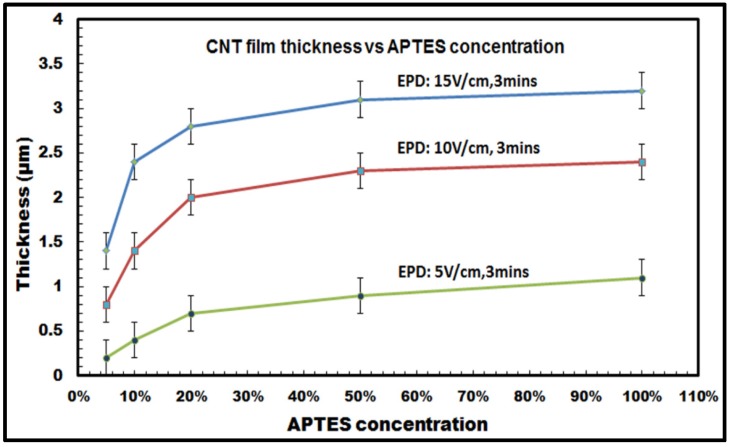
Thickness of CNT films deposited on silicon samples with varying APTES concentration at different applied electric field (E-field: 5 V/cm, 10 V/cm and 15 V/cm) for a constant deposition duration of 3 min.

It was observed that for constant deposition time and voltage, the thickness of the CNT film deposited on the silicon samples followed an increasing trend with increasing APTES concentration. As shown in Figure 8, the film thickness of the films increases from 1.4 (±0.2) µm with 5% APTES concentration to 3.3 (±0.2) µm with 100% APTES concentration for the highest E-field: 15 V/cm and 3 min of deposition duration. It can be also seen from the graph that there is a distinct increase in the film thickness as the concentration increases from 5% to about 20%, whereas the values tend to get saturated as the concentration ranges from 50% to 100%. This is more pronounced for the results obtained with 15 V/cm and 10 V/cm for 3 min of deposition duration, e.g., in Figure 8 for the E-field: 10 V/cm and 3 min of deposition duration, the thickness increases by ~2.5 times (from 0.8 to 2 µm) when APTES concentration varies from 5% to 20%, whereas it barely increases from 2.3 µm with 50% APTES concentration to 2.4 µm with 100% APTES concentration. The saturated values can be attributed to the reduction of inter-electrode electric field strength as the amount of low conducting organosilane increases on the silicon surfaces with 50% to 100% APTES concentration. The reduction in inter-electrode electric field affects the electrophoretic mobility of the CNTs, which eventually decreases the influx of the migrating nanotubes towards the deposition surface and, thus, slackens down the overall deposition process. Also, for a specific APTES concentration used in the EPD experiments, it has been also noticed that the film thickness increases with the increasing voltage (or electric field) with a constant deposition time of 3 min. Figure 8, thus, conclusively establishes the versatility of the electrophoretic deposition process, where the thickness of the deposit on the target surfaces can be precisely controlled in relation to the deposition parameters, e.g., inter-electrode electric field, degree of surface functionalization, duration of deposition.

### 2.3. Electrical Resistivity

**Figure 9:**
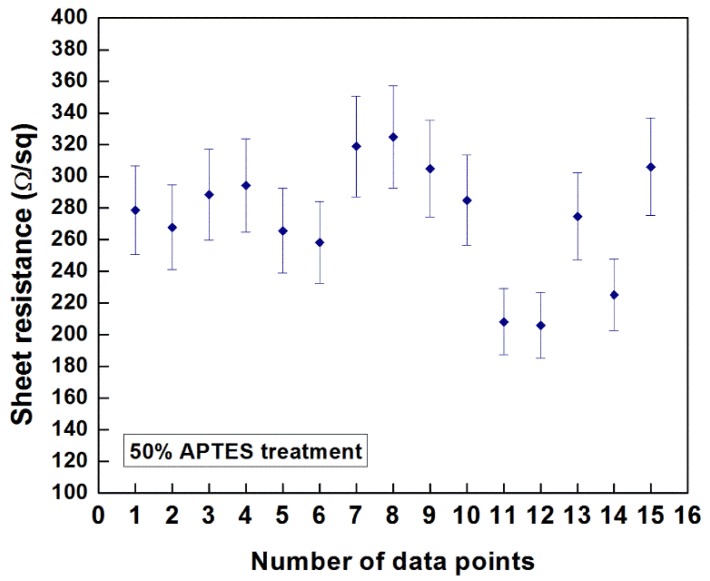
Sheet resistance of the EPD-fabricated CNT film on 50% APTES treated silicon sample deposited at an E-field of 10 V/cm for 3 min.

Preliminary investigations on the measurement of the sheet resistance of the EPD-fabricated CNT films were conducted using the four-point probe technique. It has been reported that multiple factors, e.g., the chirality of the nanotubes, defects during the synthesis and post-synthesis deposition methods, degree of dispersion, presence of residual surfactant and CNT morphology (nature of nanotube agglomeration in the deposit, packing density), influence the measured values of sheet resistance of CNT films fabricated by different coating methods. Figure 9 indicates the resistance values obtained at 15 different data points of the CNT film deposited on 50% APTES treated silicon sample at an E-field of 10 V/cm for 3 min. of deposition duration (thickness: ~2.25 µm). The measured values ranged from 205 Ω/square to 304 Ω/square (average value: ~273.83 Ω/square) with a standard deviation of 36 Ω/square. As reported by Geng *et al*. [45] and Parekh *et al*. [46], different post-deposition processing techniques, e.g., treatment with inorganic acids and thionyl chloride (SoCl_2_) with controlled exposure, can be performed to improve the conductivity of the fabricated films. A detailed research study to optimize the resistance values of the fabricated CNT films for different potential applications is being presently pursued in our research group.

## 3. Experimental Section

The experimental procedure is divided into four sections: (a) preparation of stable CNT suspensions in IPA (CNT-IPA) and water (CNT-water) for the deposition process; (b) preparation of APTES (Linear chemical Formula: C_9_H_23_NO_3_Si) solution with varying concentrations; (c) substrate preparation; and (d) EPD process.

### 3.1. Preparation of CNT Solution in IPA and Water

**Figure 10:**
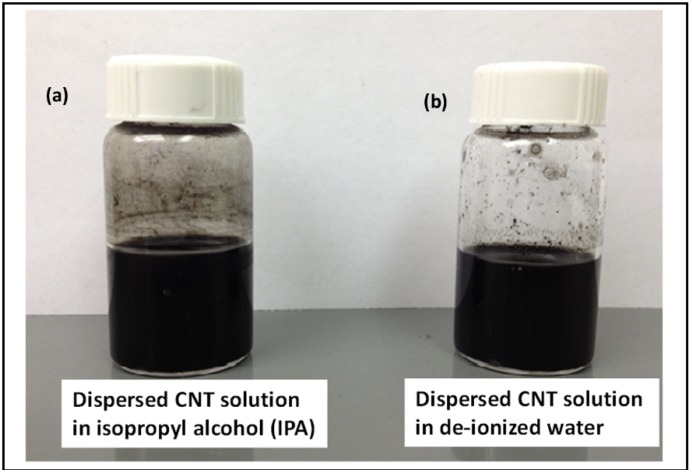
Dispersed CNT solution used for EPD experiments (**a**) stable suspension in isopropyl alcohol (CNT-IPA) and (**b**) stable suspension in water (CNT-water).

The procedure for preparing stable CNT suspension begins with acid refluxing of 100 mg of as-purchased multi-walled CNTs (purity: >98%, average wall thickness: 7–13 graphene layers, dimension: 6–13 nm (OD) × 2.5–20 µm (length), CVD, Sigma Aldrich, St. Louis, MO, USA) in 40 mL concentrated sulfuric (H_2_SO_4_) and nitric (HNO_3_) acid (volume ratio = 3:1, respectively). The solution was vigorously stirred for about 15 min with a magnetic spinner and then heated at 120 °C for 30 min on a hot plate. The acid-heat treatment of the solution produced a black slurry, which was cooled for 1 h in the fume hood. The acid-refluxed solution was then carefully washed with deionized (DI) water (18.2 MΩ-cm) and filtered in medium retentive filter papers (pore size: ~11 µm) until the resulting solution indicated pH 7 (neutral). The final black filtration residue was collected with a laboratory spatula and divided into approximately 2 equal parts. One part of it was mixed with 50 mL of isopropyl alcohol (IPA) and the other one with 50 mL of DI water. A second round of repetitive washing and filtering was performed subsequently. Both the solutions were then placed inside an ultrasonicator bath for 2 h to obtain stable CNT suspensions in IPA (referred to as CNT-IPA) and water (referred to as CNT-Water). The dispersed CNT solutions were then kept inside a chemical hood for 48 h to examine the solution stability. No visual signs of agglomeration were noticed in both the solutions and, therefore, were deemed suitable for the subsequent EPD process. Figure 10a,b shows the CNT dispersed solutions in IPA and water, respectively.

### 3.2. Preparation of APTES Solution with Varying Concentration

Different APTES solutions with varying concentration, *i.e.*, 5%, 10%, 20% and 50%, were prepared with appropriate dilution recipes in DI water and 95% ethanol. For example, 2 mL of APTES were mixed with 2 mL of DI water and 6 mL of ethanol solution for preparing 20% APTES solution. Similarly 5%, 10% and 50% APTES solution were prepared with proper dilution ratios in water and ethanol.

Silicon wafers (resistivity: 0–100 Ω-cm) were used for all the experiments. The wafers were cleaved into ~1 × 0.5 × 0.055 cm^3^ samples and were treated in warm piranha solution (H_2_SO_4_:H_2_O_2_ = 1:1) for 25 min. The samples were then immersed into 10 mL of different APTES solutions (concentrations varied as 5%, 10%, 20% and 50%) for 15 min. For 100% APTES treatment, the samples were directly immersed into an as-purchased 10 mL APTES solution without any dilution for 2 min. Following the incubation period, all the samples were rinsed in DI water to remove the excess APTES. The rinsed samples were dried in nitrogen and kept in the oven at 80 °C for 1 h.

### 3.3. EPD Process

**Figure 11:**
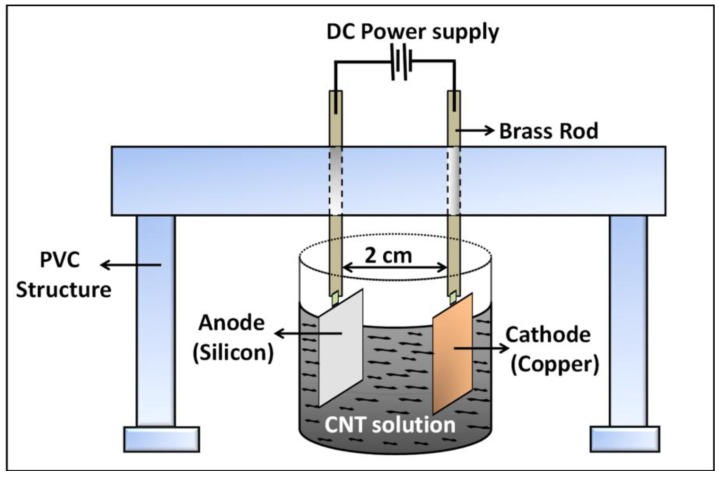
Schematic diagram of the electrophoretic deposition set-up.

A series of electrophoretic deposition experiments from CNT-IPA and CNT-water solutions was performed in a custom built set-up (as shown in Figure 11) with an APTES functionalized silicon sample as the anode and a copper plate (dimension: 1.5 × 1 × 0.2 cm^3^) as the cathode. The inter-electrode distance was fixed at 2 cm for all the experiments. Before each set of deposition experiment, both the CNT solutions were subjected to bath sonication for 15 min to minimize the amount of agglomerated CNT in the suspension. After the deposition process, the samples were dried overnight at room temperature. To investigate the effect of APTES concentration on the deposition results, EPD experiments were performed systematically with varying APTES concentration (5%, 10%, 20%, 50% and 100%) with varying deposition voltage of 10–30 V (or varying electric field (E-field) of 5–15 V/cm) for a constant deposition time for 3 min.

## 4. Conclusion and Future Work

Electrophoretic deposition of acid refluxed carbon nanotubes from CNT-IPA and CNT-water suspension has been successfully performed on silicon substrates with self -assembled APTES surface functionalization. The deposited film quality and surface properties were observed to be dependent on the suspension medium of EPD, with deposition from IPA-CNT suspension exhibiting the best results. The benefits of the electrophoretic deposition technique as a fast, reliable and reproducible room temperature coating process over the dip coating have also been established throughout the report. The electrophoretic deposition results obtained by –NH_2_ terminated APTES surface functionalization display significant difference in terms of improved homogeneity and film quality in contrast to the non-polar –CH_3_ functionalization by the HMDS treatment. Scanning electron microscopy and Raman spectroscopic results, which remained unaffected with varying APTES concentration, confirmed the presence of MWCNTs in the deposited film. The work represents significant achievement in comparison to our previous EPD results on semiconductor substrates, which showed the binding role of the metal role in the deposition scheme. The present work eliminates the need of metal incorporation in the deposition model and relies on relatively quick and inexpensive hydrophilic surface functionalization followed by EPD with remarkable deposition results by EPD. Through a series of systemic studies, we have achieved excellent and reproducible results with 20% surface functionalization between 10 and 15 V/cm for 3 min of deposition.

As regards to potential future work in this direction, we have preliminary results in extending the surface functionalization technique in an attempt to deposit CNTs on insulator substrates (e.g., silicon dioxide and silicon nitrides) by electrophoretic deposition without major variations in the deposition setup. Another potential future work in this EPD coating technique would be to increase the adhesion strength of the deposited CNTs on the semiconductor substrates, which, at this moment of our research, is being pursued by the addition of different additives in the EPD suspension. Research endeavors are also directed in the detailed investigation of the surface roughness of the electrophoretically deposited films and their application as viable substrates for Surface Enhanced Raman Spectroscopy (SERS) of different analytes. Our results in this communication confirm that the solution-based, low cost, electrophoretic deposition technique of carbon nanotubes on semiconductor substrates with –NH_2_ terminated surface functionalization has immense application potential in nano-electronics assisted by organic self-assembly, CNT-based device integration with existing CMOS technology on different semiconductor substrates and next generation hybrid electronics.
